# Neuroendocrine Tumor of the Cystic Duct: A Rare and Incidental Diagnosis

**DOI:** 10.7759/cureus.1755

**Published:** 2017-10-06

**Authors:** Fatima Ayub, Muhammad W Saif

**Affiliations:** 1 Internal Medicine, Rawalpindi Medical College, Rawalpindi, Pakistan; 2 Hematology/Oncology, Tufts Medical Center

**Keywords:** neuroendorine tumor, cystic duct, cholelithiasis, gallbladder, cholecystectomy, carcinoid tumor

## Abstract

Neuroendocrine tumors (NETs), also known as carcinoid tumors of the extrahepatic biliary tree, are extremely rare malignancies and account for 0.2% to 2% of all carcinoids of the gastrointestinal tract. The most common sites include NET in the common bile duct (CBD), followed by the perihilar region, cystic duct, and common hepatic duct (CHD). A review of the literature showed only eight cases of NETs of the cystic duct and we, hereby, present the ninth case of NET of the cystic duct in a 50-year-old woman. This was discovered incidentally after she underwent a laparoscopic cholecystectomy for her symptomatic gallbladder stones. The etiology is not known but the NETs are thought to arise from argentaffin cells (Kulchitsky cells) in the gastrointestinal tract or respiratory tract. It is possible that the rarity of these in this region may be explained by the very limited number of Kulchitsky cells there. Most of the patients are clinically asymptomatic, and the diagnosis is mostly made during surgery performed for other indications. Therefore, early recognition should be sought by differentiating the primary duct carcinoma of the bile or the cystic duct, if suspected or shown on the imaging studies because of the different therapeutic options and prognosis.

## Introduction

Adenocarcinoma is the most common neoplasm >80% arising from the epithelium of the biliary tree. Other tumor types found in the extrahepatic bile ducts include adenoma, leiomyoma, lipoma, paraganglioma, and granular cell tumor. Neuroendocrine tumors (NETs), also called carcinoid tumors of the extrahepatic bile ducts, are extremely rare, accounting for 0.2% to 2% of all the carcinoids of the gastrointestinal tract [1]. The estimated incidence has been reported between one and eight per 100,000 [1]. The literature review suggests an increased incidence of extrahepatic carcinoid tumors in younger female patients [1-2], with the most common anatomic location being the common bile duct, followed by the perihilar region and the cystic duct [2]. The scarcity of this type of tumor of the extrahepatic bile ducts makes the diagnosis even more challenging. Most of the cases reported were sporadic with no discernible underlying factor [1,3].

Due to a small number of reported cases, the natural history and pathologic and other characteristics of these neoplasms are not well understood at present, but the oncology community has adopted the World Health Organization (WHO) staging system for NETs in this location as well, which is based on tumor size, histological differentiation, Ki-67 immunostaining, invasion of adjacent tissues, and vascular and perineural invasion [3]. This staging divides these tumors into three subtypes (Table 1).

**Table 1:** WHO classification of NETs WHO: World Health Organization

CATEGORY	EXPLANATION
Well-differentiated or benign	These are both clinically and radiographically silent.
Low grade but well- differentiated	They have a good overall prognosis if surgically excised well in time before they progress.
High grade and poorly differentiated	They have the worst prognoses and are the most aggressive tumors.

However, the literature review indicated that a majority of the cases of carcinoid tumors of the extrahepatic biliary tract were usually of low malignant potential and had a favorable overall prognosis following aggressive surgery. Because of the rarity of cystic duct NET, we report here a case of a carcinoid tumor of the cystic duct and a review of the literature.

## Case presentation

**Figure 1:**
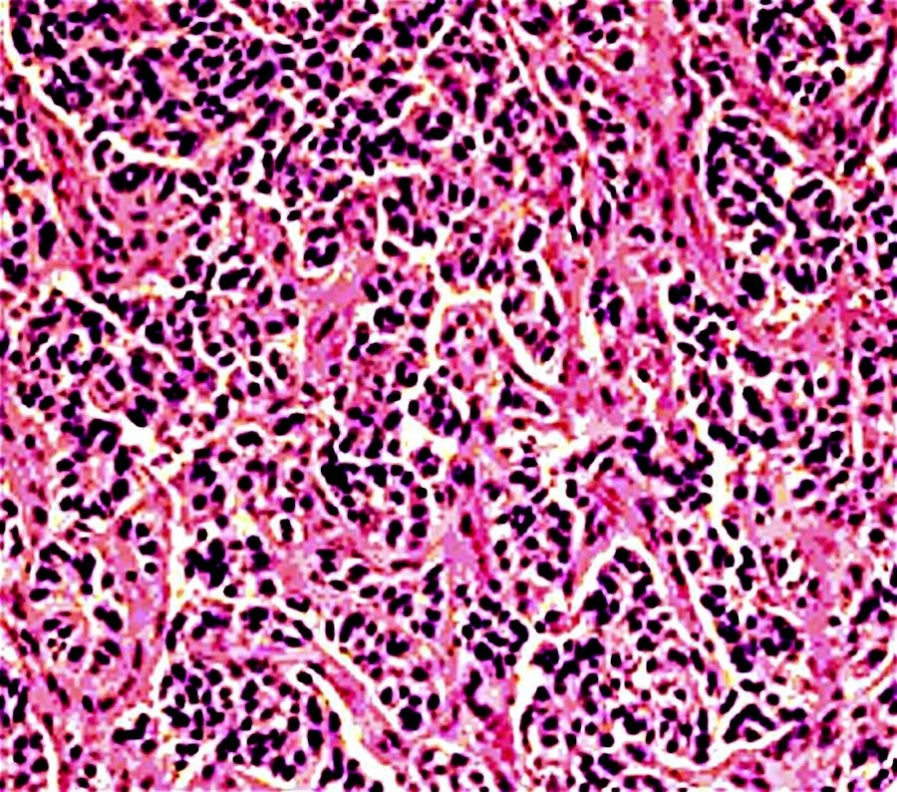
Histopathology of the cystic duct carcinoma The figure shows cords and nests of small cells on the hematoxylin and eosin stain.

A 50-year-old African American woman presented to our office for a follow-up visit for a NET of the cystic duct, which was an incidental finding post-cholecystectomy. She previously visited the emergency department (ED) with the chief complaint of right upper quadrant (RUQ) pain that was sudden in onset, and she rated it eight on a scale of one to 10. It was also associated with nausea, vomiting, and a low-grade fever. Her ultrasound revealed multiple gallstones with mild inflammatory changes in the gallbladder. After a pre-operative course of antibiotics, she underwent elective cholecystectomy, and the specimen was sent to the pathology department for assessment. It revealed a microscopic focus of NET in the resected margin of the cystic duct with perineural infiltration and raised the suspicion for lymphatic vessel invasion too (Figure 1). There was no intrinsic gallbladder tumor seen.

The patient denied any weakness, fatigue, weight loss, rashes, flushing, bowel changes, palpitations, or dyspnea, and she was doing well with no major complaints. Her past medical and surgical history included migraines, hyperparathyroidism secondary to hypocalcemia, cholelithiasis, and a NET, and apart from her recent cholecystectomy, she also had a hysterectomy for her uterine fibroids. The only medications that she was taking were daily vitamin C and vitamin D3 supplements. She was a nonsmoker and occasionally had alcoholic beverages. Family history showed that her mother died of cervical cancer.

The physical examination showed a well-developed and well-nourished woman in no apparent distress. There was no appreciable lymphadenopathy or scleral icterus. The abdomen was soft and non-tender, with no obvious hepatosplenomegaly, masses, guarding, or rebound. The rest of the examination was normal too. Her body mass index (BMI) was 29.6. She was vitally stable. The results of her liver function tests (LFTs) and tumor markers are summarized below (Table 2).

**Table 2:** LFTs and tumor markers LFTs: Liver function tests AST: Aspartate aminotransferase (normal range: <40 IU/L) ALT: Alanine aminotransferase (normal range: <55IU/L) LDH: Lactate dehydrogenase (normal range: <280 IU/L) Alkaline phosphatase (normal range: <150 IU/L) Total bilirubin (normal range: <1.2 mg/dL) Total protein (normal range: <8.3 mg/dL) CEA: Carcinoembryonic antigen (normal range: <3 ng/mL) CA 19-9: Carbohydrate antigen 19-9 (normal range: <37 U/mL) Gastrin (normal range: <600 pg/mL) Serotonin (normal range: <200 ng/mL)

LIVER FUNCTION TESTS	
AST	17 IU/L
ALT	17 IU/L
LDH	202 IU/L
Alkaline phosphatase	76 IU/L
Total bilirubin	0.30 mg/dL
Total protein	8 gm/dL
TUMOR MARKERS	
CEA 5.3 ng/mL	
CA 19-9 <3 U/mL	
Gastrin 262 pg/mL	
Serotonin 143 ng/mL	

The diagnosis of the NET was confirmed by an immunohistochemical stain for synaptophysin. The tumor stained positive only for somatostatin receptor 2A. The immunohistochemical stain for serotonin was indeterminate because of the nuclear staining artefact. A computed tomography (CT) scan showed normal liver contours with no masses or hepatomegaly and a surgically absent gallbladder. No intra- or extra-hepatic duct dilatation was appreciated. The rest of the imaging, including the bone scan, was normal with no evidence of metastatic pockets. Her screening colonoscopy was normal too.

The departments of surgery and oncology were involved in the case and decided to closely monitor the patient with three monthly follow-ups since she was asymptomatic and the initial laboratory data and imaging did not reveal anything alarming. She was followed regularly with serial chromogranin A, gastrin, cancer antigen 19-9 (CA 19-9), carcinoembryonic antigen (CEA), serotonin, pancreatic polypeptide, and vasoactive intestinal peptide (VIP) levels, respectively.

The patient is currently stable since the past two years from the time of surgery and remains free of cancer.

## Discussion

Neuroendocrine tumors (carcinoid) are thought to arise from the argentaffin cells (Kulchitsky cells) that are present throughout the gastrointestinal tract but exist in scarcity in the extrahepatic biliary tree. NETs of the biliary tract are extremely rare. The first case of a NET of the gallbladder was reported by Lubarsch in 1888 [4]. In 1961, the first carcinoid tumor that arose clearly from within the common bile duct (CBD) was reported [5]. Our review of the literature revealed less than 70 isolated reports of primary bile duct NETs with less than 10 arising from the cystic duct [6]. The incidence is relatively higher in women and no obvious underlying causes are known.

The rare incidence of NETs in the cystic duct can be explained by the very limited number of the embryonal neural crest cells in this region [7]. It is usually sporadic but the chronic inflammation of the biliary tract, for example, secondary to pancreatitis or cholecystitis, causes an increase in the number of these cells and the resulting intestinal metaplasia subsequently leads to the development of these tumors. This could have been the underlying cause in our patient too, secondary to cholecystitis.

Despite the limited number of cases reported, these NETs have been well described in the literature, ranging from benign and well-differentiated tumors to malignant neoplasms with erratic growth and aggressive behavior. Though secondary to a small number of reported cases, the natural history and pathologic, immunohistochemical, and structural features of these neoplasms are not well understood at present, and the majority of the cases reported have been described to be well-differentiated (low grade) [1-2,4,6]. Our patient had well-differentiated NET too.

The review of the previously published medical literature also revealed that most of the patients were clinically asymptomatic, similar to our patient. This is problematic because this delays the diagnosis as the majority of the cases are diagnosed following surgery for other medical conditions. However, some reported cases did have symptoms such as painless jaundice or abdominal (RUQ) pain, possibly due to the increased size of the tumor and/or impingement of the ducts [4-6]. Nonetheless, our patient did not exhibit any signs and symptoms, as it was a localized tumor. It is also important to note that just like other NETs, these tumors can also produce hormones and chemical mediators, such as serotonin, gastrin, and chromogranin-A. These functional tumors can manifest as symptoms such as flushing, diarrhea, shortness of breath, palpitations, wheezing, coughing, and hyper or hypoglycemia. Despite this wide spectrum of symptoms, which are somewhat nonspecific, a majority of the reported cases were not diagnosed preoperatively, similar to our patient. A majority of these patients were discovered as incidentalomas while having surgical explorations for other indications, at autopsy, or during the pathologic analysis of an organ removed. 

There are various diagnostic methods used to establish the diagnosis and staging of NETs, including CT scans, magnetic resonance imaging (MRI), immunohistostaining with tumor markers, endoscopic ultrasound (EUS), percutaneous cytology, somatostatin receptor scintigraphy, and positron emission tomography (PET) [4,8]. The diagnosis is confirmed with histopathology using the WHO staging described earlier [3].

Surgical resection is the treatment of choice for the cystic duct NETs that offers a potential cure [9-10]. Patients, especially those who fall in WHO category two should always get the tumor resected because they are slow growing and pose a limited potential for both local and metastatic spread. These favorable attributes mandate an early diagnosis and an aggressive surgical approach to prevent their progression to biliary obstruction and culmination into category three. The most curative surgical approach is an extrahepatic bile duct excision with portal lymphadenectomy and Roux-en-Y biliary reconstruction. But the choice of surgery varies from case to case and, sometimes, a partial hepatectomy or cholecystectomy may also be required. Adjuvant therapy is still under investigation. Although 5-fluorouracil and streptozocin have shown some activity against the gastrointestinal carcinoids, their use is still controversial [9]. Some symptomatic patients have got relief from octreotide, which is a serotonin receptor inhibitor [10]. Our patient did not need chemotherapy, as the tumor was low grade, localized, and indolent. We, therefore, found it best to follow him closely and kept surgery as an option in case it progressed with time.

## Conclusions

NETs of the extrahepatic biliary tree, and especially of the cystic duct, are very rare tumors with only a handful of cases reported in the literature. It is prudent to differentiate them from the non-neuroendocrine tumors of the same region because the therapeutic options and prognoses differ in both instances. It is also important to categorize the tumors according to the WHO classification once the diagnosis is made, either pre-operatively or post-operatively, as the decision of surgery versus medical or palliative management is based upon the category in which the tumor falls. Our patient had a well-differentiated NET of the cystic duct, which was found following cholecystectomy, and she was asymptomatic with normal radiological imaging and tumor markers. Since her tumor was localized and small, we, therefore, devised regular follow-ups with serial tests to monitor the tumor’s hibernating state, catch its possible progression or metastasis early, and save the patient from unnecessary invasive surgeries.
